# Mental health and Crohn's disease: evaluating depression through a case-referent study

**DOI:** 10.1590/1806-9282.20250570

**Published:** 2025-10-17

**Authors:** Juan Manuel Escudero Prados, Antonio Cortés-Rodríguez, Marta Elena Losa-Iglesias, Juan Gómez-Salgado, Ricardo Becerro de Bengoa Vallejo, Miguel Ángel Saavedra-García, Daniel López López, Ana María Jiménez-Cebrián

**Affiliations:** 1 Universidade da Coruña, Campus Industrial de Ferrol, Faculty of Nursing and Podiatry, Department of Health Sciences, Research, Health and Podiatry Group – Ferrol, Spain.; 2 Universidad Rey Juan Carlos, Faculty of Health Sciences – Madrid, Spain; 3 Universidad de Huelva, Faculty of Labour Sciences, Social Work and Public Health, Department of Sociology – Huelva, Spain.; 4 Universidad Espíritu Santo, Safety and Health Postgraduate Programme – Guayaquil, Ecuador.; 5 Universidad Complutense de Madrid, Faculty of Nursing, Physiotherapy and Podiatry – Madrid, Spain.; 6 Universidade da Coruña, Department of Physical Education and Sport, Group of Research in Sport Science (INCIDE) – A Coruña, Spain.; 7 University of Málaga, Department of Nursing and Podiatry – Málaga, Spain.; 8 Instituto de Investigación Biomédica de Málaga (IBIMA) – Málaga, Spain.

**Keywords:** Inflammatory bowel disease, Depression, Anxiety, Mental health, Case-control study

## Abstract

**OBJECTIVE:**

The aim of this study was to compare the frequency and severity of depressive symptoms between adults diagnosed with Crohn's disease and a demographically matched group of healthy individuals. Depressive symptoms were assessed using the Beck Depression Inventory, a validated self-report instrument.

**DESIGN AND SETTING:**

The study employed a comparative and exploratory case–control design to evaluate whether patients with Crohn's disease report significantly higher levels of depressive symptomatology than healthy controls. Patients were recruited from various regions in southern Spain.

**METHODS:**

A total of 84 participants were included, comprising 42 individuals with Crohn's disease and 42 matched controls. Assessment of depressive symptoms was performed using the Beck Depression Inventory, a validated instrument for quantifying depression severity. As the data did not meet normality assumptions, non-parametric methods were applied for statistical analysis.

**RESULTS:**

Crohn's disease patients demonstrated significantly higher depression scores compared to controls (Beck Depression Inventory: 15.55±9.99 vs. 5.76±4.18; p<0.001). The prevalence of depression (Beck Depression Inventory ≥10) was 32.1% in the Crohn's disease group versus 9.5% in the control group. Notably, severe depression was observed exclusively among Crohn's disease patients, highlighting the substantial psychological burden associated with the disease.

**CONCLUSION:**

These results highlight the significant psychological burden associated with Crohn's disease, with patients experiencing higher rates and greater severity of depression compared to healthy individuals.

## INTRODUCTION

Crohn's disease (CD) is categorized as a chronic inflammatory bowel disorder, marked by transmural inflammation that can involve any region of the digestive tract, spanning from the oral cavity to the anal region^1^. Its etiology is multifactorial, involving a complex interplay of genetic, immunological, and environmental factors, along with gut microbial dysbiosis^2^.

CD has its highest incidence between 20 and 30 years of age, frequently manifesting with clinical features including chronic diarrhea, abdominal discomfort, persistent fatigue, and unintended weight loss^3^. It should be recognized as a systemic disease that extends beyond the gastrointestinal tract, given that multiple patients exhibit extraintestinal manifestations (EIMs)^4^.

EIMs affect multiple organs and systems, strongly contributing to morbidity and, in some cases, mortality^5^. As many as 47% of patients diagnosed with inflammatory bowel disease manifest at least one EIM during disease evolution, with an increased prevalence in CD (35%)^5^. EIMs can emerge before, during, or once the intestinal disease has been diagnosed, and their presence enhances the probability of developing additional manifestations^4,6^. Among the most common EIMs are musculoskeletal manifestations, with a prevalence of up to 40%, including peripheral and axial arthritis^7^.

In recent years, the relationship between chronic inflammation and mental health has received increasing attention. It has been proposed that neuroinflammation, mediated by microglial activation and dysregulation of blood–brain barrier permeability, may contribute to the development of psychiatric conditions in chronic inflammatory disorders, including CD^8^.

The incidence of CD in Europe exhibits significant geographical variability, with rates ranging from 0.5 to 10.6 cases per 100,000 person-years, being highest in Northern Europe compared to Southern and Eastern regions, and with a prevalence of 322 cases per 100,000 person-years. This disparity likely reflects environmental, genetic, and diagnostic accessibility factors. As reported by Burisch et al.^9^, the substantial economic burden of CD, with direct healthcare costs estimated at thousands of euros per patient annually, where hospitalizations—driven by complications such as strictures and fistulae—account for up to 63% of total expenditures. Indirect costs, including lost productivity (3–6 weeks per patient per year) and disability pensions (affecting 20% of patients), further amplify the socioeconomic impact. These findings underscore the urgent need for early therapeutic interventions and targeted healthcare policies to reduce hospitalizations and improve long-term patient outcomes^10^.

Accordingly, this study aims to compare the frequency and severity of depressive symptoms between adults diagnosed with CD and a demographically matched group of healthy individuals. We hypothesize that patients with CD will exhibit significantly higher levels of depressive symptoms compared to matched healthy controls.

## METHODS

### Sample design

For this study, a total of 84 participants were recruited and divided into two groups: 42 individuals diagnosed with CD and 42 healthy controls. Patients with CD were enrolled through patient associations located in Jaén, Cádiz, Granada, and Málaga—representing various regions of southern Spain. Healthy controls were selected from an outpatient clinic. Recruitment took place between December 2023 and December 2024, using a sequential, non-randomized sampling method.

The eligibility criteria required participants to be 18 years of age or above, to have received a CD diagnosis from a gastroenterology specialist, to demonstrate ambulatory capacity, and to have provided formal authorization through the completion of the informed consent document.

The exclusion criteria for this study were designed to ensure the reliability of self-reported data and the validity of comparisons between groups. Specifically, participants were excluded if they met any of the following conditions: (1) a current diagnosis of an autoimmune disorder in the control group or (2) the presence of cognitive impairment, severe neuropsychiatric conditions, or legal disability that could interfere with their ability to comprehend study instructions or independently provide written informed consent. These conditions were evaluated during an initial clinical interview conducted by a single trained researcher to maintain consistency in data collection.

### Sample size calculation

To estimate the required sample, G*Power 3.1.9.3 software was utilized to conduct a family-wise test using a t-test for mean differences between two independent groups. The specified parameters included a one-tailed test, an effect magnitude (d) of 0.5, an α error of 0.05, a statistical power (1-β) of 0.7, and a distribution ratio between the two groups set at N2/N=1. Consequently, the minimum necessary sample for this study was determined to be 78 participants (with 37 in each group). Ultimately, the final sample comprised 84 individuals, divided into 42 patients diagnosed with CD and 42 healthy controls.

### Procedure

All assessments were conducted by a single trained researcher to ensure consistency in data collection. The study gathered a range of sociodemographic and anthropometric variables, including age, sex, weight, height, and body mass index (BMI), as well as employment status, educational level, and marital status.

In addition, all study subjects completed the BDI^11^, recognized for its effectiveness in assessing depressive symptoms^12^. This instrument consists of 21 items, each scored from 0 to 3 points, with a total possible score of 63 points. The results are categorized into different ranges: absence of depression (0 to 9 points), mild depression (10 to 15 points), moderate depression (16 to 23 points), and severe depression (24 to 57 points).

The BDI stands out for its high reliability, demonstrated by a Cronbach's alpha coefficient spanning from 0.85 to 0.889, making it suitable for both psychiatric and non-psychiatric patients, distinguishing between depression subtypes, and differentiating depression from anxiety^13^.

### Ethical considerations

This investigation was approved by the Ethics Board for Experimental Research at the University of Málaga, located in Málaga, Spain, under the reference number 137-2023-H.

### Statistical analysis

The analysis of demographic and social data included variables including age, height, weight, and BMI, as well as other independent variables. These were expressed as the mean and standard deviation (SD), with the highest and lowest values also provided. The Kolmogorov-Smirnov test was applied to assess the normality of distributions, with p>0.05 indicating a normal distribution. In this study, p-values were <0.05, suggesting deviations from normality. Accordingly, the non-parametric Mann-Whitney U test was used to identify statistically significant differences between groups.

For qualitative variables, data were expressed as frequencies and percentages. All statistical analyses were performed using SPSS software v27.0.1.0.

## RESULTS

The obtained data exhibited a non-normal distribution (p<0.05) across all analyzed variables, including age, weight, height, BMI, and BDI scores. The study was conducted with a sample of 84 subjects, divided into two groups: N=42 for the case group and N=42 for the control group. Participants in both groups were matched based on sex, BMI, and age (see Table 1).

**Table 1:** Descriptive data.

		Total (n=84) Mean±SD (min–max)	CD (n=42) Mean (SD)	Control (n=42) Mean (SD)	p-value
Age (years)		46.98±14.45 (18–76)	47.20±15.20 (18–76)	46.80±13.90 (23–67)	0.989†
Weight (kg)		72.02±15.21 (48.0–115.0)	71.5±16.70 (48.0–115.0)	72.50±13.70 (53.00–104.00)	0.528†
Height (m)		1.65±0.08 (1.48–1.85)	1.60±0.10 (1.50–1.82)	1.7±0.10 (1.48–1.85)	0.518†
BMI (kg/m^2^)		26.23±4.92 (18.06–38.87)	26.20±5.30 (18.07–38.87)	26.20±4.60 (19.72–38.67)	0.844†
Gender‡	Male	24 (28.6%)	12 (28.6%)	12 (28.6%)	N/A
	Female	60 (71.4%)	30 (71.4%)	30 (71.4%)	
Time since CD diagnosis (years)		N/A	14.35±14.58 (0.25–55.0)	N/A	

BMI: body mass index; SD: standard deviation; CD: Crohn's disease; N/A: not applicable. In all analyses, p<0.05 (95%CI) was considered statistically significant.

† The Mann–Whitney U test was applied.

‡ Frequencies (percentages).

As shown in Table 2, there was a statistically significant difference (p<0.05) in BDI scores between the two groups.

**Table 2:** Relationship between Beck Depression Inventory scores and classification in patients with Crohn's disease compared to the control group.

Outcome measurements		Total group Mean±SD (range) n=84	CD group Mean±SD (range) n=42	Control group Mean±SD (range) n=42	p-value
BDI category*	No depression	57 (67.9%)	19 (45.2%)	38 (90.5%)	N/A
	Mild	11 (13.1%)	7 (16.7%)	4 (9.5%)	
	Moderate	14 (16.7%)	14 (33.3%)	0 (0%)	
	Severe	2 (2.3%)	2 (4.8%)	0 (0%)	
BDI scores		10.65±9.06 (0–40)	15.55±9.99 (1–40)	5.76±4.18 (0–16)	**<0.001** †

* BDI: Beck Depression Inventory; SD: standard deviation; CD: Crohn's disease. Frequency, percentage (%) were employed. The BDI score classifications were defined as follows: (1) 0–9 points indicating no depression, (2) 10–15 points for mild depression, (3) 16–23 points for moderate depression, and (4) 24–63 points for severe depression.

† The Beck Depression Inventory (BDI) scores were evaluated based on the median, interquartile range, and full range (minimum to maximum). The Mann-Whitney U test was employed for analysis, with statistical significance set at p<0.05 and a 95%CI (indicated in bold). N/A: not applicable.

In the BDI categories, it can be observed that in the control group, only 4 out of 42 subjects have moderate depression, and the remaining 38 do not have depression. However, in the CD group, up to 23 individuals have some degree of depression.

## DISCUSSION

This study aims to compare the frequency and severity of depressive symptoms between adults diagnosed with CD and a demographically matched group of healthy individuals. The findings indicate that 32.1% of the patients assessed experienced depression at one or more of the three evaluated severity levels, in contrast to 9.5% among the healthy control. These findings are consistent with previous studies on CD patients that employed the BDI as a measurement tool^14^ and exceed the rates reported in studies utilizing alternative assessment methods^15^. In the study by Cho et al., 31.9% of patients with CD exhibited clinically significant levels of depression^14^, a result consistent with the conclusions of Fernández et al., who identified a strong association between the presence of systemic manifestations and elevated depression levels as assessed by the BDI^16^.

A study conducted in 2010 revealed that both genders experienced comparable levels of depression in the presence of systemic disease, although the severity was greater in men^16^. In contrast, other studies—including Cho et al.^14^—have reported a higher prevalence of depression among women. This finding suggests that women diagnosed with CD exhibit a higher predisposition to depression compared to their male counterparts, a trend also observed in the general population^17^. However, in the present study, depression severity showed no significant differences between male and female participants.

Similar to other chronic conditions such as fibromyalgia, Parkinson's disease, hemophilia, and multiple sclerosis, studies have shown that patients with these diseases experience higher depression levels relative to the general population. Furthermore, depression not only exacerbates the symptoms of the disease but also leads to a decline in patients’ overall well-being^18-20^.

This study presents several limitations. First, the relatively low participation rate among patients with CD. Second, the sample exhibited a gender imbalance, with a higher proportion of female participants. This may have introduced bias in the evaluation of psychological and clinical variables. Third, the use of a non-random, convenience sampling method reduces the external validity of the study. Furthermore, the reliance on a single self-report instrument—the BDI—without the inclusion of a structured psychiatric interview limits the ability to establish formal clinical diagnoses. While the BDI is a validated and widely used tool for assessing depressive symptomatology, it is not a diagnostic instrument.

To address the limitations identified in this study, future research should implement strategies aimed at increasing patient participation, enhancing the use and diversity of assessment tools, and ensuring more balanced and representative samples in terms of size and demographic characteristics.

## CONCLUSION

Patients with CD exhibited higher depression scores compared to the healthy population. These results highlight the psychological vulnerability of this population and reinforce the need for integrating regular mental health screening into clinical care.

## Data Availability

The datasets generated and/or analyzed during the current study are available from the corresponding author upon reasonable request.
